# Nanopore Sequencing for Epigenetic Decoding: Applications, Analytical Constraints, and Translational Prospects

**DOI:** 10.3390/ijms27209175

**Published:** 2026-10-16

**Authors:** Jiachao Xin, Jiaxuan Liu, Yichao Liu, Xinru Liu, Mengke Ma, An Huang, Zijie Ye, Pengfan Zhang, Xue Wang, Tao Zeng, Xiumei Lin, Chengwei Liu, Liang Lu, Liang Chen

**Affiliations:** 1 Key Laboratory for Enzyme and Enzyme-like Material Engineering of Heilongjiang, College of Life Science, Northeast Forestry University, Harbin 150040, China; xinjiachao@nefu.edu.cn (J.X.); liujiaxuan@nefu.edu.cn (J.L.);; 2 State Key Laboratory of Genome and Multi-Omics Technologies, BGI Research, Hangzhou 310030, China; 3 Key Laboratory of Spatial Omics of Zhejiang Province, BGI Research, Hangzhou 310030, China

**Keywords:** nanopore sequencing, epigenetics, multi-omics, precision medicine, DNA modification, RNA modification, biomarkers

## Abstract

Epigenetic regulation involves multiple molecular layers, including DNA and RNA modifications, histone post-translational modifications (PTMs), and chromatin accessibility. These processes contribute to cell identity, development, and disease, but their measurement remains technically challenging because sequence context, molecular modification, chromatin state, and transcript structure are often assayed separately. As a representative third-generation sequencing technology, nanopore sequencing is profoundly reshaping the interpretation of the epigenetic code through its distinctive capabilities, including long-read sequencing, direct single-molecule signal detection, and the identification of nucleic acid modifications without bisulfite conversion or PCR in appropriate workflows. This review summarizes applications of nanopore sequencing in epigenomics and epitranscriptomics and evaluates emerging nanopore-based approaches for protein and histone sensing. We focus on what can currently be measured, the experimental and computational requirements for reliable decoding, and the limitations that must be addressed before broader multi-omic and clinical use.

## 1. Introduction

**Figure 1:**
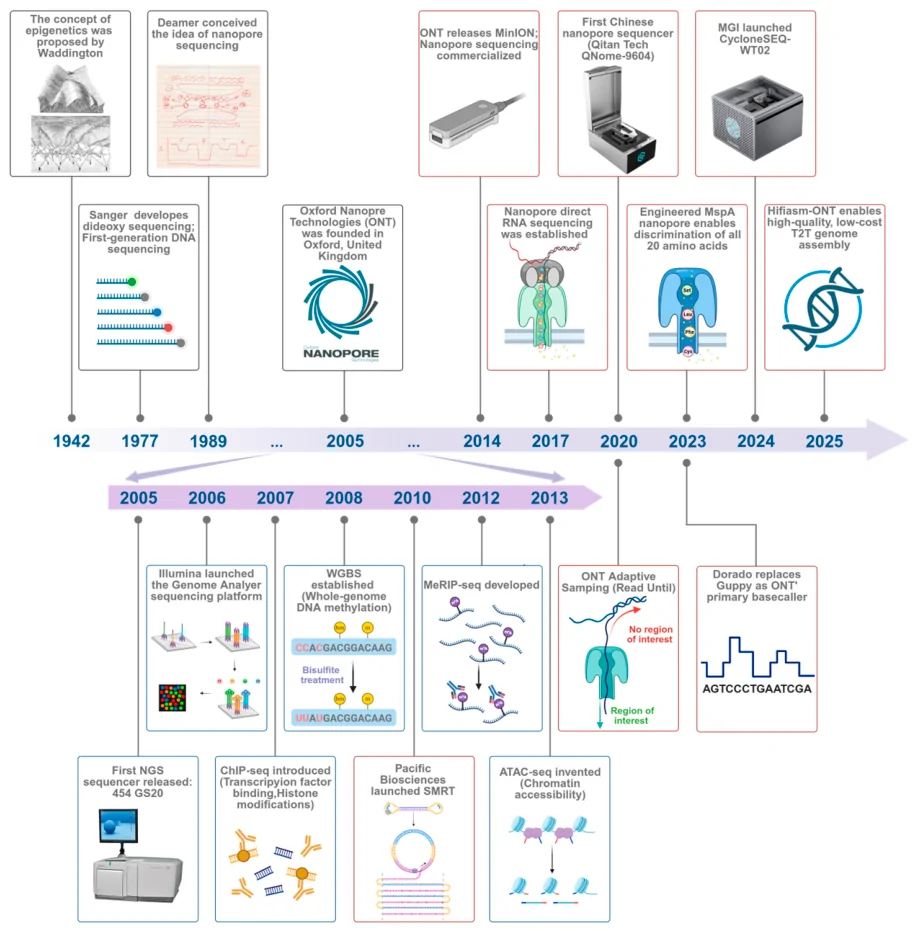
Development history of nanopore sequencing and epigenetic technologies. A chronological overview spanning foundational molecular biology discoveries (1942–1970s), the advent of first-generation Sanger sequencing (1977), the proliferation of second-generation high-throughput sequencing (NGS) alongside landmark epigenetic profiling tools (e.g., ChIP-seq, WGBS, MeRIP-seq, and ATAC-seq), and the paradigm shift driven by third-generation single-molecule nanopore sequencing (from initial commercialization to native DNA/RNA modification mapping, full-length transcriptomics, and experimental histone/protein nanopore sensing). Created in BioRender. Han, L. (2026). https://BioRender.com/qs1sm43.

In 1977, Sanger and co-workers pioneered the dideoxy chain-termination method—the first efficient, accurate, and scalable DNA sequencing technology [1]. Beginning in 2005, the advent of second-generation, high-throughput sequencing platforms—exemplified by Roche and Illumina—substantially alleviated the limitations of first-generation technologies, including low throughput, high cost, and long turnaround times, while increasing throughput and reducing per-base costs [2]. Empowered by this technological revolution, a diverse array of epigenetic profiling methodologies was rapidly established and widely adopted across the biomedical sciences (Figure 1). In 1942, Conrad H. Waddington originally coined the term “epigenetics” to describe the developmental mechanisms through which a genotype unfolds into distinct phenotypes [3]. Today, epigenetics, encompassing DNA methylation, RNA methylation, histone modifications, and chromatin remodeling, orchestrates transcriptional activity and genome stability, thereby profoundly governing pivotal biological processes, including organismal development, disease pathogenesis, and aging [4,5,6]. Leveraging next-generation sequencing (NGS) platforms, a sophisticated repertoire of epigenetic profiling methodologies has been established, epitomized by whole-genome bisulfite sequencing (WGBS) [7,8], chromatin immunoprecipitation sequencing (ChIP-seq) [9,10], assay for transposase-accessible chromatin with sequencing (ATAC-seq) [11], and methylated RNA immunoprecipitation sequencing (MeRIP-seq) [12]. These approaches usually rely on chemical conversion, antibody-based enrichment, enzymatic reactions or restriction digestion to detect specific epigenetic marks [13]. While highly useful, these traditional assays have several technical drawbacks. The complex sample preparation can degrade nucleic acids and erase the original sequence context [14]. Furthermore, short-read sequencing often fails to accurately map allele-specific modifications or resolve signals in repetitive genomic regions [15]. Another major limitation is that most existing methods only capture one type of epigenetic mark per assay. This inability to read multiple modification layers simultaneously with the primary genomic sequence makes it difficult to study complex epigenetic regulatory networks.

Unlike traditional assays that rely on amplification, native nanopore sequencing enables the direct, single-molecule interrogation of native nucleic acids [16,17]. Because it bypasses destructive chemical treatments such as bisulfite conversion, this approach preserves DNA integrity [14]. By decoding raw ionic current fluctuations via machine learning, the platform simultaneously profiles multiple epigenetic marks [18]. Furthermore, the ultra-long reads generated by this platform effortlessly resolve highly repetitive genomic regions and complex structural variants [19,20]. For RNA studies, direct RNA sequencing eliminates reverse transcription biases, offering a reliable method to profile full-length transcript isoforms alongside their native epitranscriptomic marks [21]. Ultimately, these capabilities make nanopore sequencing highly effective for discovering biomarkers in complex diseases [22] and unraveling epigenetic mechanisms during development [14], providing a promising platform for integrative epigenetic research.

Recent reviews tend to focus on single dimensions—such as computational tools for DNA methylation or isolated clinical values—yet epigenetic regulation actually operates as a complex, multi-layered molecular network [23,24,25]. Furthermore, nanopore sequencing itself is an integrated system that deeply couples biotechnology with informatics. Currently, a comprehensive perspective that connects these multiple epigenetic layers, and specifically bridges the gap between analytical limitations and actual clinical translation, remains scarce. To fill this gap, our manuscript briefly outlines the rapidly evolving landscape of epigenetic technologies and examines the diverse epigenetics applications of nanopore sequencing. More importantly, we critically assess the existing bottlenecks rooted in both sequencing chemistry and computational algorithms. By evaluating these technical advances alongside current challenges, we aim to provide a practical framework to help transition nanopore-based epigenetic analysis from basic research into clinical implementation.

## 2. Overview of Nanopore Sequencing Technology

**Figure 2:**
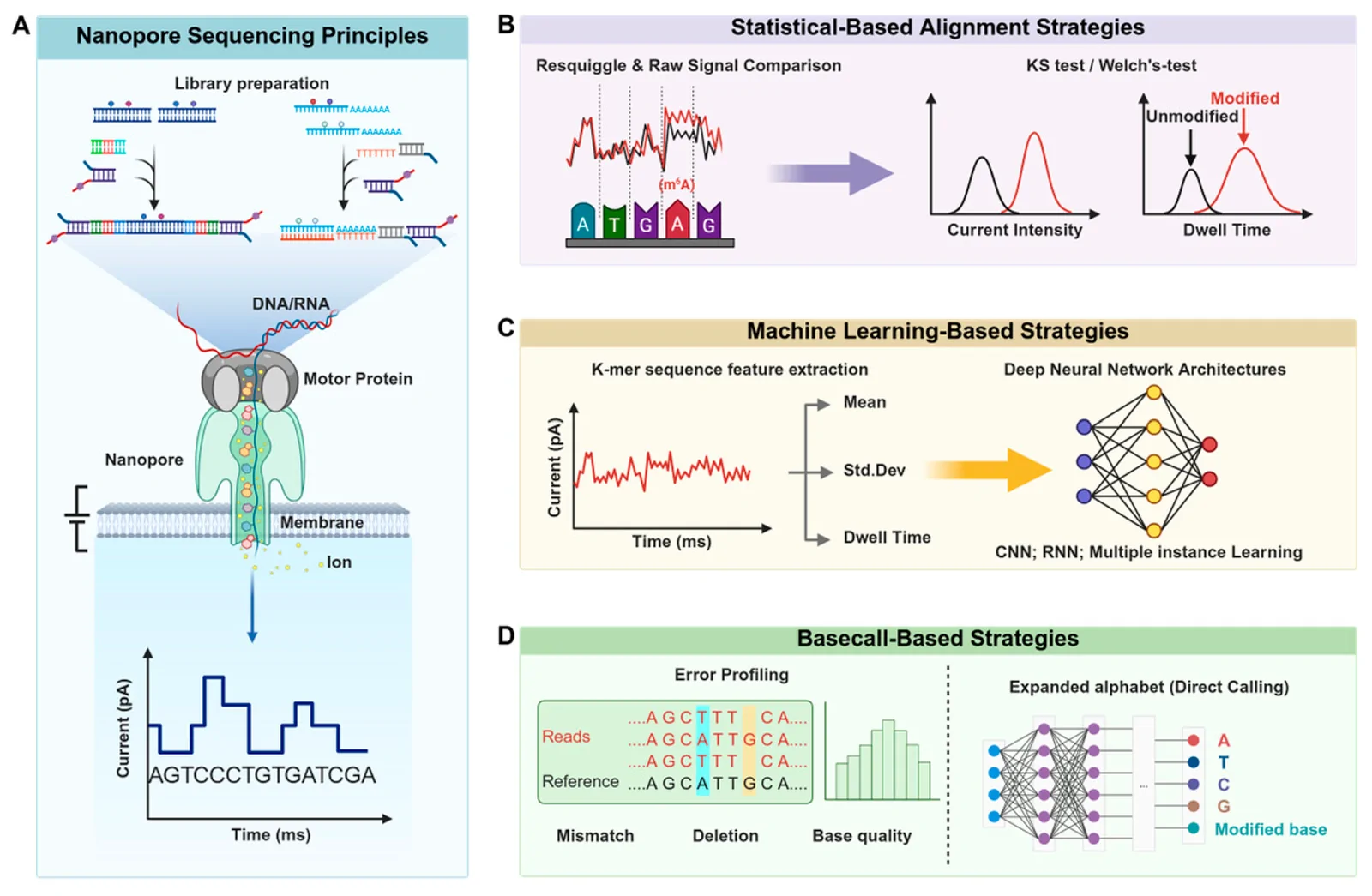
Principles of nanopore sequencing and strategies for modification detection. (**A**) Working principle of nanopore sensing: Native DNA or RNA molecules are prepared and ratcheted through a nanoscale protein pore embedded in a synthetic lipid bilayer by a motor enzyme. As consecutive nucleotides translocate through the pore constriction, they generate characteristic, time-dependent ionic current modulations (pA) that correspond to specific k-mer sequences. (**B**) Direct comparative analysis of normalized and rescaled ionic current distributions between native samples and unmodified/in vitro transcribed (IVT) or PCR-amplified controls using statistical metrics to detect modification-induced current shifts. (**C**) Extraction of k-mer level ionic current features coupled with deep neural network architectures to achieve high-accuracy, de novo modification classification. (**D**) Basecalling-derived and extended-alphabet strategy: Modification detection via comparative error profile analysis or direct deconvolution using extended-alphabet basecallers that simultaneously decode canonical (A, C, G, T/U) and modified bases. Created in BioRender. Han, L. (2026). https://BioRender.com/v2izef0.

Nanopore sequencing, a representative single-molecule long-read technology, can address several limitations of conventional epigenetic assays through long reads and native-signal analysis, although performance remains dependent on sample preparation, pore chemistry, and computational models [16,19]. Fundamentally, this technology relies on trans-membrane electrical sensing. A constant voltage applied across a lipid bilayer drives an ionic current, while a motor protein regulates the stepwise translocation of single-stranded DNA or RNA through the pore (Figure 2A). As the nucleic acid passes through the pore, the multiple nucleotides and their modifications occupying the channel constriction (termed k-mer) induce distinctive ionic current disruptions based on their chemical structures and pore interactions. These minute current disruptions are captured in real time via low-noise amplifier circuitry and subsequently deciphered by advanced machine-learning basecalling algorithms, simultaneously decoding primary nucleotide sequences and epigenetic modifications (Figure 2) [16,26,27,28].

The concept of nanopore sequencing was initially proposed by David Deamer in 1989 [29]. This theoretical framework was later experimentally validated in 1996 by Kasianowicz et al., who provided the first proof-of-principle demonstration that single-stranded DNA translocating through an α-hemolysin pore produces characteristic ionic current blockades [30]. Subsequent technological breakthroughs across single-base resolution [31,32], translocation rate control [33,34], and engineered pore design [35,36] firmly established the basis for the commercialization of nanopore sequencing platforms. In 2014, Oxford Nanopore Technologies (ONT) unveiled the MinION, the first palm-sized portable sequencer, inaugurating a new era of commercial and field-deployable nanopore sequencing [37]. ONT has since undergone continuous generational upgrades from the early R6 to R9 pores and ultimately to the current high-accuracy R10.4.1 pore, complemented by the introduction of Kit 12 (SQK-LSK112) and Kit 14 (SQK-LSK114) DNA sequencing chemistries, alongside dedicated RNA kits such as SQK-RNA002 and SQK-RNA004 [28,38,39]. As of 2026, iterative upgrades across chemical systems, nanopore geometry, and algorithmic models have improved nanopore sequencing capabilities, with ultra-long read N50 values around 100 kb reported in optimized workflows and individual reads sometimes reaching the megabase (Mb) scale [40,41,42]. The ONT PromethION P48 can deliver multi-terabase output under specified high-throughput conditions; such maximum yields should not be interpreted as routine output for epigenetic experiments [43]. Importantly, nanopore platforms can support direct or indirect inference of multiple epigenetic marks (such as 5mC, 5hmC, and 6mA on DNA, alongside m^6^A and m^5^C on RNA), providing a platform for multi-omic epigenetic investigations [44,45].

## 3. Decoding the Epigenome via Nanopore Sequencing

**Figure 3:**
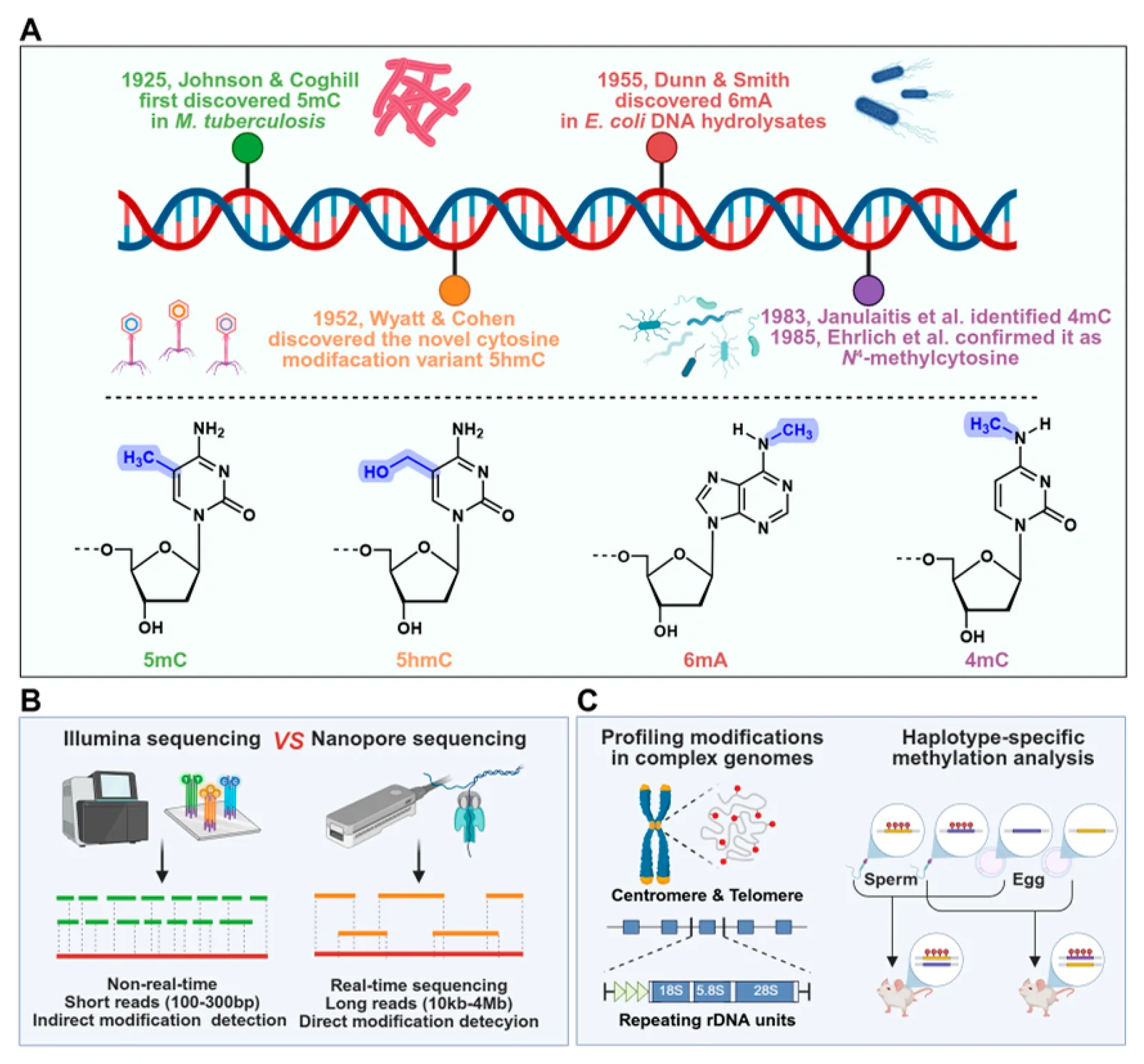
Discovery history of representative DNA modifications and applications of nanopore sequencing in epigenomics. (**A**) Historical timeline highlighting the landmark identification of 5-methylcytosine (5mC; Johnson & Coghill, 1925 [46]), 5-hydroxymethylcytosine (5hmC; Wyatt & Cohen, 1952 [47]), N6-methyladenine (6mA; Dunn & Smith, 1955 [48]), and N4-methylcytosine (4mC; Janulaitis et al., 1983 [50]; Ehrlich et al., 1985 [51]), alongside their chemical structures and methyl or hydroxymethyl substituents. (**B**) Comparison of short-read and long-read sequencing technologies. Conventional short-read platforms (e.g., Illumina) typically generate reads of approximately 100–300 bases and infer modifications through assay-specific conversion or enrichment steps; DNA damage depends on the particular protocol. In contrast, Oxford Nanopore sequencing provides long-read, real-time data acquisition, and selected native base modifications can be inferred on individual molecules without amplification in suitable workflows. (**C**) Long-read applications in complex genomic architectures and haplotype-resolved epigenomics. Left: High-resolution profiling of native epigenetic modifications across previously intractable genomic domains, including centromeric arrays, telomeres, and subtelomeric tandem repeats. Right: Single-molecule haplotype-resolved methylation profiling, enabling direct physical phasing of parental alleles to dissect allele-specific methylation (ASM), genomic imprinting, and epigenetic heterogeneities without pedigree reliance. Created in BioRender. Han, L. (2026). https://BioRender.com/yahsebv.

Decades of fundamental research have established a core set of DNA methylation marks present across both prokaryotes and eukaryotes. Following the early discovery of 5-methylcytosine (5mC) [46], researchers subsequently identified other critical modifications, including 5-hydroxymethylcytosine (5hmC) [47], *N*^6^-methyladenine (6mA) [48,49], and *N*^4^-methylcytosine (4mC) [50,51] (Figure 3A). Their abundance and regulatory roles vary across organisms: 5mC is well established in plants and mammals, whereas 6mA and 4mC are prominent in many prokaryotes. Profiling these modifications therefore requires attention to species and biological context [52,53,54]. This section specifically details the current strategies and progress in decoding these foundational DNA marks.

### 3.1. Detection Methods and Analytical Tools for DNA Modifications

Traditional profiling of 5mC relied heavily on bisulfite conversion, laying the groundwork for classic whole-genome bisulfite sequencing (WGBS) [7,8,55]. However, this approach requires harsh chemical treatments and PCR amplification, often leading to severe DNA degradation and amplification bias [56,57]. Furthermore, standard WGBS fails to differentiate 5mC from 5hmC. Profiling additional modifications therefore necessitates specialized parallel methodologies, such as oxidative bisulfite sequencing (oxBS-seq, which measures 5mC and estimates 5hmC by comparison with matched BS-seq) [58], 6mA DNA immunoprecipitation sequencing (6mA-DIP-seq) [59], and Tet-assisted bisulfite-related protocols (4mC TAB-seq) [60]. While nanopore sequencing now enables the simultaneous detection of multiple epigenetic marks from a single read, these established methods remain indispensable for orthogonal validation.

The earliest work on nanopore detection of DNA modifications centered primarily on the “fifth base”, 5mC [61]. Multiple early studies confirmed that methylated bases elicit distinct ionic current blockades during strand translocation through the nanopore, demonstrating the feasibility of native modification detection [62,63]. However, modification profiling at this stage was largely constrained to simple signal-level comparisons in the absence of dedicated algorithmic frameworks or bioinformatic tools. In 2017, the introduction of Nanoraw (v0.4.2) marked an important step in nanopore methylation data analysis [64]. It demonstrated sensitivity to 5mC, 4mC, and 6mA in tested contexts, but a differential signal alone does not determine the chemical identity of an unknown modification. Nanopolish (https://github.com/ jts/nanopolish, accessed on 28 September 2026) uses a hidden Markov model (HMM) with trained k-mer current distributions for CpG methylation calling [65]. SignalAlign (https://github.com/ArtRand/signalAlign, accessed on 28 September 2026) combines a variable-order HMM with hierarchical Dirichlet process models and evaluates 5mC, 5hmC, and 6mA in defined settings [66]. By contrast, DeepSignal (https://github.com/bioinfomaticsCSU/deepsignal, accessed on 28 September 2026) uses convolutional and recurrent neural networks for 5mC and 6mA detection [67]; the published DeepMod2 models target 5mC in CpG contexts [26]; and DeepPlant targets plant 5mC in CG, CHG, and CHH contexts [68]. These tools differ in their supported modifications, chemistry, and training data, so capabilities should not be inferred from the model architecture alone. ONT workflows have also integrated modification inference with basecalling: Guppy (https://nanoporetech.com/document/Guppy-protocol, accessed on 28 September 2026) and Dorado (https://github.com/nanoporetech/dorado/, accessed on 28 September 2026) provide modification-aware models, whereas Megalodon (https://github.com/nanoporetech/megalodon, accessed on 28 September 2026) uses model outputs and reference alignment to generate modification calls [18]. Consequently, methylation detection no longer exclusively depends on standalone downstream event-alignment tools, but directly outputs modification-tagged reads during basecalling. Table 1 summarizes the assays and the capabilities established in the cited studies.

### 3.2. Performance Analysis of DNA Modification Detection Tools Based on Nanopore Technology

Nanopore-based computational tools employ divergent signal processing strategies, causing significant variations in sensitivity, specificity, and false-positive rates (FPR) [69,70]. Rigorously benchmarking these epigenetic callers is thus critical for their reliable application in clinical and mechanistic research. In modification detection, sensitivity reflects the correct identification of methylated sites, whereas specificity measures the exclusion of unmethylated ones. Systematic evaluations indicate a pervasive trade-off between these two metrics across existing nanopore tools [70,71]. Traditional algorithms like Nanopolish often exhibit limited sensitivity because they apply strict double cutoffs to discard ambiguous reads [65,71]. Conversely, deep learning models significantly enhance performance by extracting higher-order features from raw currents. DeepSignal, for example, utilizes CNNs and RNNs to achieve superior genome-level accuracy on both human and *E. coli* datasets [67]. However, this sensitivity gain frequently comes at the expense of an inflated FPR. Whole-genome evaluations show that while leading tools (e.g., Nanopolish, Megalodon, DeepSignal, Guppy) perform well globally, their accuracy deteriorates in challenging contexts, such as discordant adjacent CpGs, low-CG-density zones, and repetitive sequences [72]. This heterogeneity largely stems from training data bias. Since most models are trained on specific synthetic datasets, their generalization capacity drops when encountering novel sequences, leading to misclassification [18,72]. To counter this, taxon-specific tools like DeepPlant (https://github.com/xiaochuanle/DeepPlant, accessed on 28 September 2026) integrate tailored neural architectures. By accommodating the high repeat content and unique methylation patterns of complex plant genomes, such specialized models offer greater robustness and lower FPRs than generic alternatives [68].

Importantly, these performance conclusions are primarily drawn from cross-sectional comparisons under specific sequencing chemistries, coverage thresholds, and reference conditions. Existing benchmarking studies still lack consensus regarding ground truth definitions, site-filtering criteria, and evaluation metrics [71,72]. Moving forward, it is imperative to establish a standardized framework encompassing diverse species, modification types, sequence contexts, and pore versions. Furthermore, coupling nanopore calls with orthogonal validations—such as bisulfite sequencing (BS-seq) or oxidative bisulfite sequencing (oxBS-seq)—will be critical to ensuring reliable tool assessment and downstream biological interpretation.

### 3.3. Profiling DNA Methylation Patterns in Complex Genomic Regions

Owing to short read lengths and sequence alignment challenges, NGS-based epigenetic assays often have difficulty resolving complex genomic loci enriched in high repetitiveness, heterozygosity, and structural variations (Figure 3B). As a result, challenging regions such as telomeres, centromeres, ribosomal DNA (rDNA), tandem repeat arrays, and large transposable elements have long remained diagnostic blind spots in methylation profiling. This bottleneck has substantially hindered the systematic exploration of whole-genome epigenetic regulation [17,73].

In human rDNA research, nanopore sequencing has enabled single-copy methylation profiling of 45S rDNA tandem repeat clusters, facilitating analysis beyond the limitations of some conventional assays [74]. This work revealed that non-coding regions of human rDNA are heavily hypermethylated while roughly half of the coding units are unmethylated, and demonstrated that higher rDNA copy numbers correlate with increased methylation within coding regions [74]. In human genomics, the Telomere-to-Telomere (T2T) Consortium combined Oxford Nanopore ultra-long reads and PacBio HiFi sequencing to construct the complete genome reference T2T-CHM13 from a haploid complete hydatidiform mole cell line, closing the ~8% unresolved sequence gap in GRCh38 [75]. Concurrently, researchers from Johns Hopkins University capitalized on the long-read capability and direct modification profiling of nanopore sequencing to map single-base methylation across classic “genomic dark regions” such as telomeres and centromeres, contributing to a methylome map of the CHM13 human genome [76].

Centromere protein A (CENP-A, designated CenH3 in plants) is the pivotal epigenetic determinant of centromeric identity, function, and inheritance [77]. DiMeLo-seq (Directed Methylation with Long-read Sequencing), pioneered by Altemose et al., enables genome-wide protein–DNA interaction profiling, significantly advancing CENP-A studies across repetitive regions [78,79]. Integrating nanopore sequencing with DiMeLo-seq revealed an association between CENP-A occupancy and hypomethylated centromeric regions flanked by methylated heterochromatin; the causal contribution of these boundaries requires further validation [80,81]. Furthermore, pronounced centromeric hypomethylation has been identified in malignancies such as ovarian cancer, showing particular elevation in HRD subtypes and providing novel epigenetic biomarkers for tumor stratification [82]. Owing to long read lengths and simultaneous native modification detection, nanopore sequencing has improved the characterization of repetitive regions relevant to selected genetic diseases. Giesselmann et al. established STRique, a CRISPR-Cas targeted enrichment method, proving that repeat copy numbers can be accurately quantified at the single-molecule level in a PCR-free manner, while defining crucial correlations between DNA methylation alterations and short tandem repeat (STR) expansions in *C9orf72* (GGGGCC repeats) and *FMR1* (CGG repeats) [83]. Concurrently, Stevanovski et al. utilized ONT ReadUntil (adaptive sampling) to perform targeted STR sequencing and synchronous DNA methylation profiling for diverse neurological and neuromuscular disorders within a single research sequencing workflow [84].

In plant complex genomes, single-molecule targeted accessibility and methylation sequencing (STAM-seq) enables concurrent detection of chromatin accessibility and DNA methylation within highly repetitive regions (HRRs) [85]. Using STAM-seq in *Arabidopsis*, researchers discovered low chromatin accessibility and CHH methylation at telomeres, contrasted with elevated DNA methylation in subtelomeric domains [85]. Furthermore, telomere and subtelomere characterization in the green alga *Chlamydomonas reinhardtii* identified three major subtelomeric elements: Sultan, Subtile, and Suber [86]. Following chromosomal rearrangements, relocated ancestral Sultan elements maintain high methylation, while newly inserted sequences near telomeres gain elevated methylation levels [87]. Developments in machine learning enhance nanopore sequencing’s modification detection accuracy, enabling comprehensive epigenetic profiling across complex genomic regions (Figure 3C).

### 3.4. Profiling Haplotype-Specific DNA Methylation

Haplotype-specific methylation (HSM) governs critical biological processes, including genomic imprinting and allele-specific expression, making it a major determinant of development and disease [88,89]. However, profiling allele-specific methylation has been hindered by the limitations of short-read platforms. Because short fragments lack long-range phasing capabilities, they cannot reliably distinguish parental alleles or map epigenetic marks to distinct haplotypes [14,15]. In addition, bisulfite-induced DNA fragmentation further destroys long-range epigenetic connectivity, impeding mechanistic studies of haplotype-resolved methylation [14].

Haplotype phasing approaches fall into three main classes: pedigree-based phasing (related individuals), population linkage disequilibrium (LD)-based statistical phasing, and physical (read-based) phasing [90,91]. Pedigree-based phasing deduces haplotypes via Mendelian inheritance from pedigree data, but suffers from limited sample accessibility and cannot phase de novo variants [92]. Population-based LD phasing uses statistical inference guided by reference panels; however, it exhibits poor accuracy for rare variants and only resolves local LD blocks, failing to achieve chromosome-level phasing contiguity [93]. Conversely, physical phasing harnesses long sequencing reads (such as Oxford Nanopore) to directly resolve parental alleles at the physical single-molecule level (Figure 3C, right panel). In 2019, Gigante et al. first demonstrated long-read-based assignment of DNA methylation to individual haplotypes, showing that nanopore reads enable far more straightforward phasing than reduced representation bisulfite sequencing (RRBS), with 85.7% of reads assigned in the reported dataset [14]. Subsequently, Lee et al. developed nanoNOMe-seq to concurrently generate allele-specific, genome-wide maps of DNA methylation and chromatin accessibility in human cells [20]. Together, these studies illustrate the utility of nanopore long reads for methylation-aware phasing. Pedigree-free single-molecule nanopore phasing attains an SNP phasing accuracy of 99.95% [94]. Similarly, NanoStrand-seq integrates Strand-seq with nanopore reads to directly detect and phase SNPs and SVs at the whole-chromosome scale in diploid cells [95]. Moreover, MethPhaser serves as an optimal, methylation-driven phasing framework for HSM studies; it overcomes the barrier of extended homozygous tracts, refines phasing across the HLA locus and other medically relevant genes, and confirms widespread haplotype-specific methylation patterns across the human genome [96].

Capitalizing on ultra-long reads, native epigenetic detection, and high-precision phasing, nanopore sequencing serves as a pivotal platform for haplotype-specific epigenetic studies [24]. In precision oncology, ONT PromethION-based long-range phasing enables the detection of allele-specific differentially methylated regions (aDMRs) and allele-specific expression, demonstrating biallelic tumor suppressor gene inactivation in an advanced cancer cohort [97]. For facioscapulohumeral muscular dystrophy type 1 (FSHD1), ultra-long nanopore reads accurately size and phase D4Z4 repeat contractions in somatic mosaic patients and confirm pathogenic allele hypomethylation, establishing a benchmark for preimplantation epigenetic testing [98]. Epigenetic studies using mouse hybrid models demonstrate that ~7% of autosomal epigenetic marks follow non-Mendelian inheritance, governed by distal regulatory interactions, parental effects, sex, and tissue context, providing fresh insights into the transgenerational inheritance of complex diseases [99]. Addressing clinical diagnostics for imprinting disorders (IDs), Urakawa et al. recently established a targeted long-read sequencing (T-LRS) pipeline covering all imprinting-related regions [100]. This platform accurately benchmarked reference CpG methylation ranges across parent-of-origin DMRs and supported concurrent structural and epigenetic profiling in patients with multi-locus imprinting disturbance (MLID); broader clinical validation is still required [100].

## 4. Decoding the Epitranscriptome via Nanopore Sequencing

**Figure 4:**
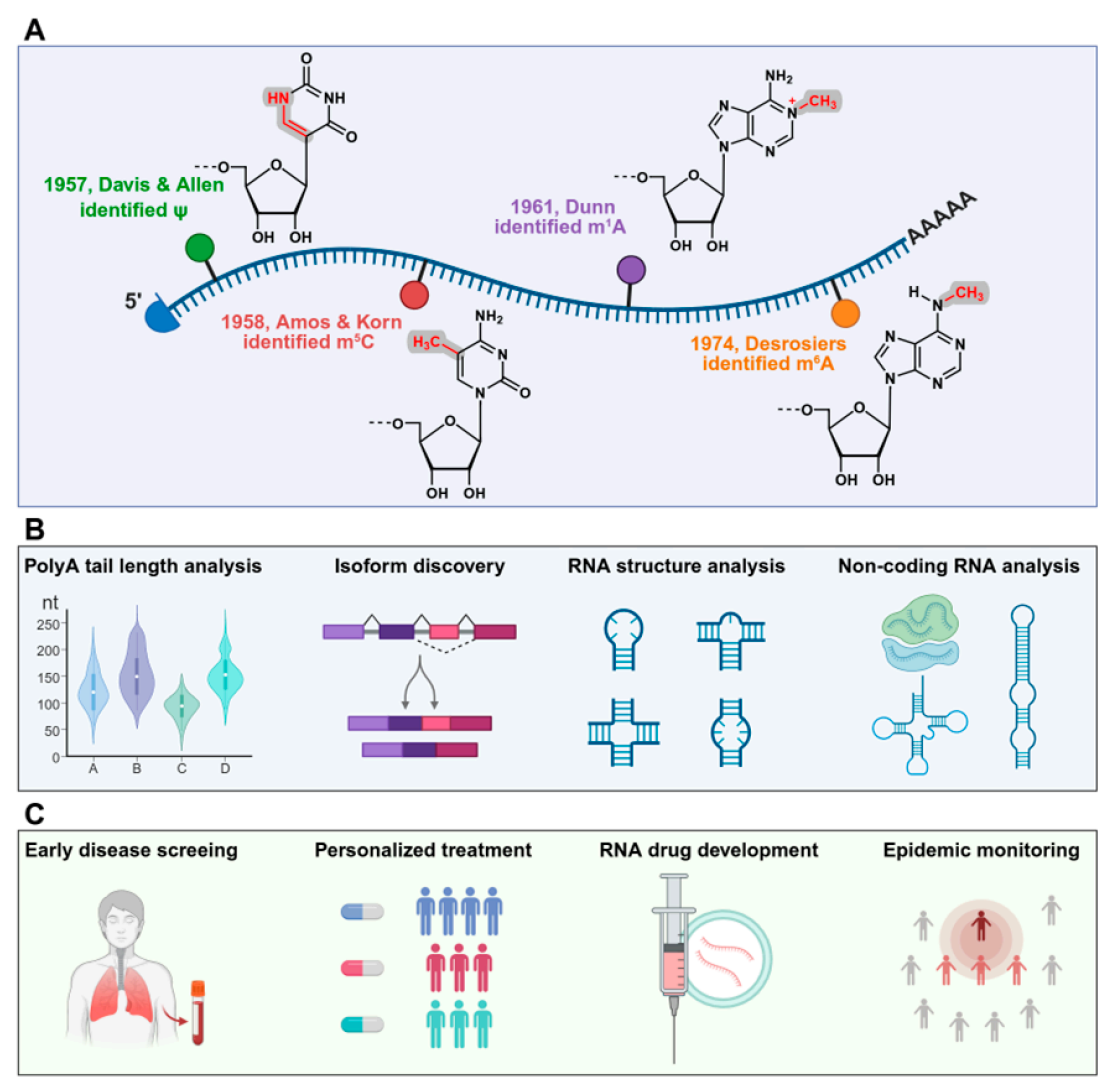
Discovery history of representative RNA modifications and applications of nanopore sequencing in epitranscriptomics. (**A**) Chemical landscapes and historical timeline of canonical RNA modifications, highlighting milestone discoveries of key epitranscriptomic marks: pseudouridine (Ψ, Davis & Allen, 1957 [101]), 5-methylcytosine (m^5^C, Amos & Korn, 1958 [102]), N1-methyladenosine (m^1^A, Dunn, 1961 [103]), and N6-methyladenosine (m^6^A, Desrosiers, 1974 [105]). (**B**) Multidimensional feature extraction from native RNA molecules. Direct RNA sequencing enables simultaneous, single-molecule resolution of multiple transcriptomic layers: (i) quantitative estimation of polyadenylation tail length distributions across biological samples; (ii) full-length transcript isoform identification and alternative splicing resolution; (iii) RNA secondary structure probing; and (iv) high-density non-coding RNA (ncRNA) profiling. (**C**) Translational, therapeutic, and clinical paradigms. The multi-omics capabilities of nanopore sequencing drive diverse downstream applications, encompassing: (i) early disease screening and liquid biopsy biomarker identification from clinical specimens; (ii) personalized medicine and patient stratification; (iii) rational design, quality control, and optimization of synthetic RNA therapeutics and mRNA vaccines; and (iv) rapid pathogen tracking and decentralized epidemic/pandemic surveillance. Created in BioRender. Han, L. (2026). https://BioRender.com/mey962y.

RNA chemical modifications act as essential epitranscriptomic marks that drive post-transcriptional gene regulation. Following the early isolation of pseudouridine (Ψ) [101] and 5-methylcytosine (m^5^C) [102], subsequent studies uncovered additional modifications with profound structural and functional impacts (Figure 4A). For instance, the characterization of 1-methyladenine (m^1^A) revealed a positively charged mark capable of disrupting Watson–Crick base pairing, thereby actively altering RNA secondary structures and protein-binding dynamics [103,104]. Likewise, *N*^6^-methyladenosine (m^6^A) was established as the most abundant internal modification in eukaryotic mRNA [105,106,107]. To date, over 170 distinct chemical modifications have been identified across various RNA species, revealing a highly complex epitranscriptome [108].

Conventional RNA modification profiling strategies based on short-read next-generation sequencing platforms have predominantly relied on antibody-mediated immunoprecipitation (e.g., MeRIP-seq [109], m^1^A-ID-seq [110]) or chemical-assisted conversion strategies (e.g., Pseudo-seq [111], RNA-BisSeq [112]), which provided transcriptome-scale maps for selected RNA marks. Nevertheless, these techniques face inherent limitations: short reads cannot capture full-length transcripts, hindering multi-modification co-localization and the identification of novel alternative splicing isoforms. Furthermore, conventional techniques typically detect only one RNA modification at a time. Profiling multiple marks therefore requires parallel library preparations, creating a significant bottleneck in epitranscriptomic research [113]. Since 2017, nanopore direct RNA sequencing (DRS) has provided a robust alternative [114]. Coupled with advanced machine-learning algorithms, DRS can simultaneously map sequences and various modification signals on unamplified single molecules.

### 4.1. Deciphering Epitranscriptomic Regulation in Coding RNAs

DRS reads native RNA strands directly, retaining modification-associated signals and avoiding PCR amplification of the sequenced RNA, thereby offering a viable, single-molecule resolution paradigm for resolving the intricate epitranscriptomic landscapes of coding RNAs [39,115,116]. In mammalian messenger RNAs (mRNAs), m^6^A and pseudouridine (Ψ) constitute the two most prevalent chemical modifications, playing cardinal roles in orchestrating mRNA stability, alternative splicing, and translation efficiency [117]. However, their single-molecule co-occurrence landscapes and combinatorial regulatory mechanisms on individual transcripts have long remained elusive. Leveraging machine-learning algorithms such as NanoSPA, researchers have achieved the simultaneous single-molecule detection of m^6^A and Ψ, uncovering a transcriptome-wide opposing co-occurrence pattern and establishing their synergistic and hierarchical regulation of translation through polysome profiling [117]. Moreover, the development of the CHEUI computational framework has enabled the single-molecule prediction of m^6^A and m^5^C from the same sequencing dataset, allowing estimation of modification-associated signal differences and site-level co-occurrence patterns [118]. Subsequent work revealed that pseudouridylation globally impacts m^6^A and m^5^C deposition; by genetically depleting the pseudouridine synthases PUS7 or DKC1 coupled with nanopore direct RNA sequencing, researchers quantitatively captured stoichiometric perturbations across all three major modification marks, establishing the cardinal role of PUS7-dependent pseudouridylation in modulating epitranscriptomic crosstalk [119]. By enabling simultaneous, multi-mark quantitative analysis without antibody capture or chemical labeling, nanopore sequencing establishes a distinct platform for deciphering complex modification networks in coding RNAs [120,121].

In addition to modification profiling, nanopore sequencing simultaneously captures complex transcript structural dynamics, encompassing alternative splicing (AS), gene fusions, and novel isoform discovery (Figure 4B) [21,122]. Conventional short-read sequencing often fails to capture intact mRNA molecules, making it difficult to resolve homologous splice isoforms. Nanopore sequencing overcomes this by generating reads that span multiple exon-exon junctions, enabling precise isoform identification [123]. For example, profiling the human frontal cortex via the PromethION platform revealed over 400 novel isoforms and identified 99 that are differentially expressed in Alzheimer’s disease [124]. By leveraging raw ionic current signatures in conjunction with specialized bioinformatic algorithms, DRS further enables the estimation of polyadenylation [poly(A)] tail length at single-molecule resolution [125,126]. In a human brain epitranscriptome study, DRS simultaneously mapped RNA modifications, isoform expression, and poly(A) tail lengths. The results demonstrated that structural features, such as 3′ UTR length, dictate m^6^A deposition, establishing a clear link between m^6^A patterning, poly(A) dynamics, and brain-region-specific isoform expression [127]. Ultimately, integrating these layers of transcriptomic data provides a comprehensive framework for interrogating post-transcriptional networks, while its biological interpretation remains dependent on experimental design and analytical validation.

### 4.2. Deciphering Epitranscriptomic Regulation in Non-Coding RNAs

Non-coding RNAs (ncRNAs), particularly tRNAs and rRNAs, are heavily modified to maintain translation efficiency and accuracy. Nonetheless, their intricate secondary structures and modification diversity have historically impeded precise global mapping [128,129]. By directly reading native RNA without PCR or chemical conversion, nanopore sequencing can improve analysis of these highly homologous and modified transcripts, although capture, alignment, and modification assignment remain challenging [130]. Targeting tRNAs—a highly abundant and diverse class of cellular ncRNAs—Nano-tRNAseq enables the simultaneous quantification of tRNA abundance and modifications in a single experiment, revealing the molecular crosstalk and interdependence among co-occurring modification types in Saccharomyces cerevisiae [131]. In rRNA modification research, the NanoConsensus pipeline significantly improves detection performance by integrating outputs from distinct calling algorithms (EpiNano, Nanopolish, Tombo, and Nanocompore). This method reported antibiotic-associated changes in modification signals at bacterial ribosomal A- and P-sites, providing novel epitranscriptomic perspectives into antibiotic mechanisms [132]. Additionally, nanopore long-read sequencing paired with advanced computational strategies, such as 1D score clustering, has been explored for detecting rare ncRNA modification-associated signals that are difficult to measure with some conventional methods [133].

### 4.3. RNA Vaccines and Viral Surveillance: Therapeutic Modification Characterization and Pathogen Tracking

mRNA vaccines deliver mRNAs encoding pathogen or tumor antigens into host cells to synthesize antigens via endogenous translation systems, inducing specific humoral and cellular immunity [134,135]. The success of these vaccines relies on incorporating modified nucleosides such as m^1^Ψ, Ψ, and m^5^C, which boost mRNA stability and translation efficiency while dampening unwanted immunogenicity [136]. Nanopore direct RNA sequencing distinguishes unmodified U from Ψ and m^1^Ψ, directly revealing T7 RNA polymerase incorporation preferences and sequence-dependent rules in in vitro transcription (IVT) reactions to guide modified nucleoside integration in mRNA vaccines [137]. With therapeutic RNAs increasingly integrating diverse chemical modifications (including base, ribose, and backbone chemistries), nanopore sequencing may provide a tool for evaluating selected therapeutic RNA modifications in native contexts, although concurrent detection of multiple non-natural modifications remains challenging (Figure 4C) [138].

Rapid and accurate pathogen detection is critical for public health surveillance. The portability and real-time output of nanopore sequencing make it a powerful platform for outbreak monitoring and on-site diagnostics. In 2015, nanopore technology was first deployed during the Ebola outbreak for emergency surveillance and pathogen identification; post-sample receipt, sequencing took only 15–60 min, with sample-to-result turnaround achieved within 24 h, greatly improving surveillance timeliness [139]. In basic virology, nanopore sequencing is widely applied not only to rapidly identify RNA virus variants, such as SARS-CoV-2, but also to directly profile chemical modifications across native viral RNA genomes, facilitating mechanistic insights into modification-driven viral immune escape and replication control (Figure 4C) [140,141].

### 4.4. Analytical Limitations in Quantitative Epitranscriptomics

Despite the unprecedented advantages of DRS and sophisticated computational frameworks, quantitative epitranscriptomic analysis remains constrained by several biological and technical confounders that directly impact result reliability.

A central challenge stems from k-mer effects. Nanopore sensors detect ionic current disruptions across a multi-nucleotide window rather than reading single isolated bases. Because a chemical modification alters the electrical signature in conjunction with its surrounding sequence context, deconvoluting these signals is inherently complex [142]. Algorithms trained on limited k-mer diversity often struggle to generalize, misclassifying modifications in underrepresented sequence contexts [143,144]. Transcript abundance and sample integrity strictly limit quantitative precision [145]. Since DRS reads native RNA without PCR amplification, low-abundance transcripts frequently fail to reach the read depth requisite for statistically robust modification stoichiometry. This depth limitation is severely exacerbated by RNA degradation. Given that native RNA sequencing typically proceeds in the 3′-to-5′ direction (captured via poly-A tails), degraded transcripts prematurely exit the pore. This directional sequencing bias disproportionately truncates 5′ ends, effectively precluding the reliable quantification of modifications in upstream regions and skewing global epitranscriptomic maps [146].

Standard DRS library preparation includes RNA denaturation followed by reverse transcription to generate RNA–cDNA hybrids for better library stability. This step can improve sequencing yield or reduce some structural impediments, but it does not establish that RNA secondary structure, degradation, or modification-dependent capture has no effect on quantification [145]. In particular, predicted modification probabilities are not automatically calibrated estimates of chemical stoichiometry. Cross-reactivity and neighboring modifications can generate false positives, and good performance on balanced synthetic controls may not transfer to low-abundance modifications in biological samples [144]. Quantitative studies therefore require modification-free controls, appropriate positive controls, sufficient read depth, and orthogonal validation where feasible [121].

## 5. Detection of Protein Post-Translational Modifications via Experimental Nanopore Sensors

Protein post-translational modifications (PTMs) refer to covalent chemical alterations occurring on amino acid residues following ribosomal polypeptide synthesis. As a fundamental mechanism shaping functional proteome diversity, more than 650 PTM types have been catalogued [147]. These modifications play vital roles in signal transduction, metabolic regulation, and cell cycle control by altering protein conformation, activity, localization, and interactions [147]. Traditional PTM detection relies heavily on mass spectrometry, which typically requires labor-intensive sample preparation, enzymatic digestion and chromatographic fractionation, and may have limited sensitivity for low-abundance modifications depending on the analytical workflow [148,149]. In specific designs, nanopore sensors operate independently of labels, antibodies, or enzymatic cleavage, providing a potential route to single-molecule detection [148,150]. Nanopore-based sensing has emerged as an experimental platform for protein and modification sensing, although routine protein sequencing and PTM-omics remain under development.

### 5.1. Technical Progress and Breakthroughs in Nanopore PTM Sensing

Phosphorylation was among the earliest PTMs detected using nanopore sensors. In 2014, the Bayley team employed α-hemolysin (α-HL) to distinguish unphosphorylated, monophosphorylated, and diphosphorylated thioredoxin variants, validating the feasibility of single-molecule nanopore sensing for PTM detection [151]. Since then, multiple research groups have successively employed different biological nanopores, most notably *Mycobacterium smegmatis porin* A (MspA), fragaceatoxin C (FraC), and cytolysin A (ClyA), to detect selected PTMs, including glycosylation, acetylation, methylation, and ubiquitination, mainly in model peptides or proteins [152,153,154]. In work published online in 2023, Huang and colleagues engineered a Ni-NTA-modified MspA nanopore to achieve the first single-molecule discrimination of all 20 standard amino acids and four representative PTMs with a machine learning-assisted accuracy of 98.6%, breaking through a critical resolution bottleneck and laying the foundation for nanopore protein sequencing [155]. In solid-state nanopore sensing, atomically thin molybdenum disulfide (MoS_2_) nanopores provide superior signal-to-noise ratios, pushing amino acid detection to sub-Dalton mass resolution and establishing the physical basis for single-residue PTM profiling [156]. Together, diverse nanopore architectures have enabled the discrimination of multiple PTM types, such as glycosylation, acetylation, methylation, and ubiquitination, as well as the accurate localization of specific modification sites [157].

### 5.2. Limitations of Nanopore Sensors in Peptide and Protein Detection

Recent years have witnessed significant progress in nanopore sensing for peptide and protein detection. Yet, this field remains far less mature than commercialized nanopore techniques for DNA/RNA sequencing. Unlike nucleic acids, which feature a relatively uniform negatively charged backbone and a smaller canonical monomer alphabet that simplify signal decoding [158,159], proteins present intrinsic technical challenges. They are assembled from 20 diverse amino acids with distinct chemical and electrical properties. Such complexity hinders precise signal recognition and makes standardized signal-to-sequence mapping highly difficult [158,159].

A critical bottleneck in this field—beyond signal decoding—is the controlled capture and directional transport of peptides/proteins through the nanopore. To address this, several methods have emerged, such as charged-tag assistance [160,161], molecular motor drives [162,163], and electroosmotic flow (EOF) regulation [164]. EOF provides a promising strategy for capture and transport. An electric field drives mobile ions and associated solvent through an ion-selective pore, generating hydrodynamic drag that can assist capture and translocation of neutral or heterogeneously charged analytes [165]. EOF can reduce reliance on a uniformly charged analyte or an added charged tag, but net motion still depends on competition with electrophoretic forces, pore-analyte interactions, and solution conditions [166]. Furthermore, the broad applicability and reliability of EOF for analyzing complex PTMs remain to be fully verified [150]. Future research must focus on optimizing nanopore geometry and electrochemical conditions, while incorporating machine learning techniques to improve analytical discrimination and reproducibility [167,168,169].

## 6. Indirect Profiling of Histone Modifications via Nanopore Sequencing

Histone post-translational modifications (HPTMs) are core epigenetic PTMs that critically regulate chromatin structure and gene expression [170,171]. Common HPTMs, such as acetylation, methylation, phosphorylation, and ubiquitination, precisely modulate vital biological processes, including cell proliferation, differentiation, senescence, and stress responses, by altering chromatin accessibility and recruiting specific regulatory proteins without changing the DNA sequence [172,173]. High-throughput and cost-effective NGS-based technologies, such as ChIP-seq and CUT&Tag, have greatly advanced genome-wide histone modification profiling and mechanistic studies [174,175]. However, inherent limitations like short read lengths and amplification biases render these methods inadequate for deeply resolving complex epigenetic mechanisms [19]. In recent years, novel methods combining third-generation long-read sequencing, such as nanoHiMe-seq, BIND&MODIFY, and scNanoSeq-CUT&Tag, address some of these technical limitations [44,176,177]. Notably, these nanopore-based strategies do not directly detect histone modifications themselves; instead, they effectively capture and read spatial proxy signals correlated with target histone modifications to achieve indirect profiling of HPTMs. NanoHiMe-seq and BIND&MODIFY utilize antibody-guided methyltransferases to label DNA adjacent to target HPTMs with exogenous m^6^A, enabling the joint analysis of DNA methylation and histone modifications, as well as the identification of allele-specific modifications and long-range interactions [44,176]. At the single-cell level, scNanoSeq-CUT&Tag merges the high sensitivity of CUT&Tag with long-read nanopore sequencing to map genome-wide chromatin modifications [177]. Together, nanopore-based epigenetic platforms provide complementary approaches for investigating links between chromatin modifications and gene regulation.

## 7. Detection of Epigenetic Biomarkers

**Figure 5:**
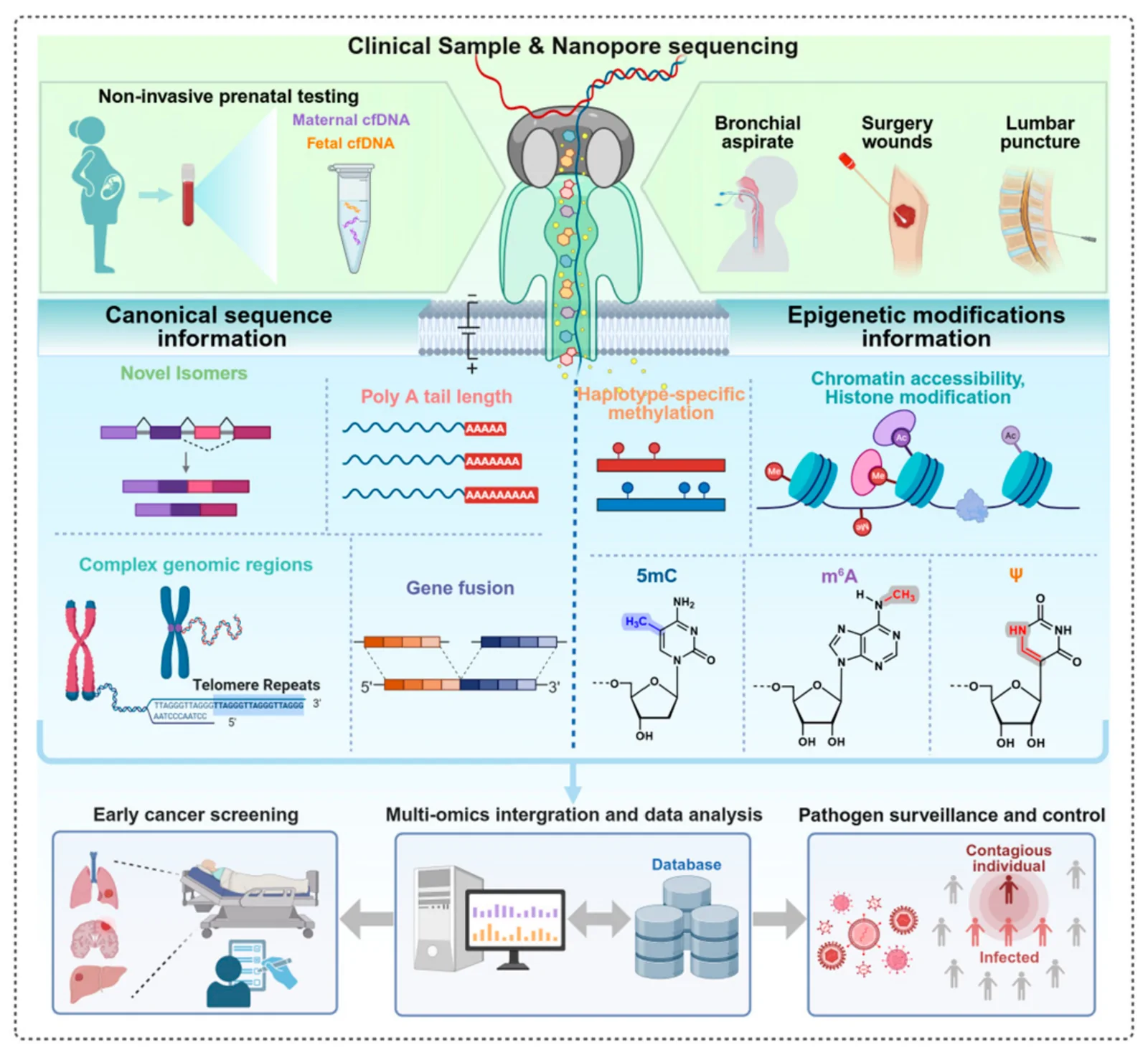
Multi-omics profiling capabilities of nanopore sequencing and its translational potential in precision medicine. Diverse clinical specimens (top) are subjected to nanopore sequencing. The panels combine distinct DNA, RNA, and chromatin workflows; they do not represent simultaneous measurement of every layer from one unprocessed specimen. Histone modifications and accessibility require dedicated indirect assays. Potential applications include prenatal testing and early cancer detection, alongside established sequence-based pathogen surveillance; clinical readiness varies by application. Created in BioRender. Han, L. (2026). https://BioRender.com/nusiffk.

Biomarkers are quantifiable molecules that objectively reflect physiological and pathological conditions, providing essential evidence for clinical diagnosis and treatment. Unlike traditional diagnostic indicators, epigenetic biomarkers (such as DNA modifications, RNA modifications, and HPTMs) offer insights into gene function while reflecting the effects of environment and lifestyle on health. They are therefore of significant clinical value for early screening, precise diagnosis, prognostic evaluation, and improving patient outcomes [178,179,180]. Compared to conventional assays, nanopore sequencing combines single-molecule long-read capabilities with signal-based modification analysis and can support joint interpretation of sequence and modification information (Figure 5). This advantage supports translational evaluation of epigenetic biomarkers [180,181]. Moreover, liquid biopsy studies have evaluated single-molecule nanopore platforms for profiling cell-free DNA (cfDNA) and circulating tumor DNA (ctDNA), although sensitivity depends on input amount, enrichment, coverage, and error control [182,183]. Furthermore, utilizing DRS, recent studies have unveiled that rRNA modification patterns exhibit distinct specificity across tissues, cell types, developmental stages, and disease states, supporting their evaluation as candidate biomarkers for tissue-of-origin identification and tumor-versus-normal classification [184]. Hao-Fountain syndrome (HAFOUS), a rare neurodevelopmental disorder caused by pathogenic variants in the *USP7* gene, can be characterized by a disease-associated DNA methylation episignature. Leveraging long-read nanopore sequencing, researchers have achieved the simultaneous detection of *USP7* genetic variants and known HAFOUS epigenetic signatures, demonstrating the feasibility of integrating genetic and epigenetic measurements in rare-disease diagnostics [185]. Additionally, phosphorylated α-synuclein has been studied as a candidate biomarker for Parkinson’s disease, although diagnostic performance and assay standardization remain concerns [186,187]. Together, these findings highlight the potential of nanopore-derived epigenetic signatures as clinical biomarkers. However, their transition into routine diagnostics will require extensive analytical and clinical validation (Figure 5).

To transform these epigenetic modifications into reliable biomarkers for clinical diagnosis, precise and reliable quantitative analysis is essential. A critical consideration for quantitative biomarker analysis is that nanopore sequencing does not uniformly capture all molecular species: differences in length, sequence context, and modification status can produce distinct capture and translocation efficiencies, such that observed read counts do not directly reflect relative concentrations [131,188]. To address this, several calibration strategies have been developed. Analyte-specific calibration curves relating input concentration to event rate offer the highest quantitative accuracy but require purified reference materials for each biomarker [189,190]. A more scalable approach involves internal standards—such as synthetic spike-ins or adaptor-embedded reference ladders (CAPTOR)—which enable cross-sample normalization with high fidelity [191,192]. Computational methods further complement these experimental strategies by correcting length-dependent biases [193]. Nonetheless, for clinical epigenetic biomarkers, particularly low-abundance analytes in liquid biopsies, rigorous validation with orthogonal methods remains essential to ensure quantitative reliability.

## 8. Future Perspectives and Translational Challenges

Nanopore sequencing enables concurrent acquisition of genomic sequences and multiple epigenetic modification signals, offering unique strengths in the decoding of epigenetic information. Nevertheless, traversing the chasm from “technological feasibility” to “clinical application” necessitates addressing formidable challenges and exploring innovative paradigms across multiple dimensions, particularly in relation to analytical precision, model performance, and multimodal data integration [39,194].

### 8.1. Nanopore Biochemical Components and Experimental Systems

Currently, the primary technical bottlenecks are the accuracy and sensitivity of modification detection. The ionic current shifts induced by epigenetic marks are often subtle and easily masked by complex background noise [195]. Additionally, methods for the efficient enrichment and nucleic acid extraction of trace samples remain limited, struggling to preserve molecular integrity while satisfying the demands of high-throughput sequencing [183]. To surmount these challenges, future efforts must focus on the engineering of novel nanopore proteins and the upgrading of sequencing reagents, alongside optimizing motor protein control mechanisms to prolong signal acquisition time, which may improve the signal-to-noise ratio [38,196,197]. Meanwhile, the development of more robust sample pretreatment technologies—such as specific enrichment strategies tailored for trace samples like ctDNA—may improve detection of low-abundance modifications [183]. Amplification-based approaches such as NanoRCS improve sequence consensus but do not preserve native DNA methylation in the amplified product [198]. For RNA, CapTrap-seq improves full-length cDNA representation but does not directly retain native RNA modification signals [199], whereas Direct-miR-seq uses custom adaptors to support direct sequencing of short miRNAs [200]. Protocol choice should therefore follow the intended molecular measurement.

### 8.2. Stability and Accuracy of Modification Detection Models

Nanopore sequencing heavily relies on algorithmic models for signal decoding. While the evolution of ONT sequencing chemistry and the shift in basecalling algorithms from Hidden Markov Models to deep neural networks have improved overall modification detection, these advancements have also introduced significant “model drift” [70,201]. This phenomenon occurs when changes in training data distributions or algorithm designs produce inconsistent modification calls for the same sample across different software versions [120]. Compared to the earlier R9.4.1 flow cells, ONT’s R10.4.1 pores offer chemistry and model improvements that can enhance modification discrimination, including 5mC/5hmC calling under validated conditions [201,202]. However, changing the pore type necessitates a complete recalibration of raw signal features. Older modification models cannot simply be applied to signal generated by newer pores; rather, they require rigorous retraining or fine-tuning to maintain detection accuracy.

Cutoff configuration serves as another decisive factor in modification detection [120]. Detection tools typically output a modification probability or confidence score per genomic site, requiring users to define a cutoff to determine the modification status. The chosen cutoff not only strikes a balance between sensitivity and specificity, but also directly impacts the accuracy of downstream biological interpretations [120]. For instance, under low-coverage conditions, a lenient cutoff can inflate false-positive rates, while strict criteria may lead to the omission of true low-frequency modifications [71].

### 8.3. Multi-Omics Bioinformatic Analysis and Clinical Translation

To advance the clinical translation of nanopore sequencing, it is essential to establish a comprehensive framework for computational reproducibility. First, metadata for model training data must be made publicly available. This not only helps trace the sources of variations in results but also provides a foundation for subsequent model updates and validation. Second, establishing benchmark datasets and standardized evaluation processes is key to addressing model drift. By constructing gold-standard datasets that encompass a wide range of biological sample types, sequencing platforms, and chemical systems, researchers can systematically evaluate the performance stability of different software versions and models [70].

Although nanopore-enabled multi-omics may support precision-medicine research, the associated bioinformatics analyses face significant hurdles. The massive single-molecule electrical data generated by long-read sequencing creates substantial demands for storage, data transfer, and parallel computing during whole-genome epigenetic mapping [27,203,204]. Additionally, the joint multi-omics analysis requires precise alignment of heterogeneous multimodal data and extraction of complex regulatory networks, while standardized frameworks remain underdeveloped [205]. Addressing these issues will require validated statistical, machine-learning, and data-management frameworks. For instance, contrastive learning-driven deep hybrid networks (e.g., NanoCon) have been reported to improve signal classification for known modification sites; performance should be assessed across independent datasets and chemistries [195]. Furthermore, unsupervised and semi-supervised strategies like 1D score-clustering may reduce reliance on extensively annotated datasets, but discovery of novel nucleic-acid modifications still requires chemical or orthogonal validation [133].

Finally, the emergence of additional sequencing platforms, including those evaluated in preliminary preprints, highlights the need for cross-platform benchmarking and standardized protocols to improve data comparability and reproducibility [206,207].

## 9. Conclusions

In summary, nanopore long-read sequencing has demonstrated utility in selected basic and translational epigenetic applications. Through ongoing technological upgrades and algorithmic breakthroughs, this platform may contribute to the investigation of complex, dynamic epigenetic networks. By deeply decoding the molecular basis of epigenetic regulation, it may support studies of disease mechanisms and biomarker development, but claims regarding early screening, prognostic subtyping, and personalized treatment require prospective clinical validation.

## Figures and Tables

**Table 1:** Comparison of NGS-based experimental assays and nanopore-based computational tools for detecting core DNA modifications.

Category	Method/Software	Target DNA Modifications	Core Principle	Major Limitations
NGS-based experimental assays	WGBS [7,8]	5mC + 5hmC (combined)	Bisulfite chemical conversion + short-read sequencing	DNA degradation; PCR amplification bias
	oxBS-seq [58]	5mC; 5hmC by paired BS-seq subtraction	Oxidation of 5hmC followed by bisulfite conversion	Paired libraries needed for 5hmC; conversion bias and DNA loss
	6mA-DIP-seq [59]	6mA	Antibody-based immunoprecipitation enrichment	Antibody specificity and contamination controls required; low spatial resolution
	4mC-TAB-seq [60]	4mC	TET oxidation of 5mC followed by bisulfite conversion; 4mC remains read as C	Complex experimental workflow; only detects 4mC
Nanopore-based computational tools	Nanoraw [64]	Modification-associated signals (tested: 5mC, 4mC, 6mA)	Comparative statistical test on ionic-current distributions (native DNA vs. amplified unmodified control)	Mandatory control sample required; suboptimal accuracy
	Nanopolish [65]	5mC in CpG contexts	Hidden Markov Model (HMM) with pre-trained k-mer current models	Limited performance for 5hmC, 6mA, 4mC
	SignalAlign [66]	5mC, 5hmC, 6mA in tested contexts	Variable-order HMM with hierarchical Dirichlet process current models	Early-stage tool; poor cross-species generalization
	DeepSignal [67]	5mC, 6mA	Convolutional and recurrent neural networks	Highly dependent on quality of training datasets
	DeepMod2 [26]	5mC in CpG contexts	Signal features with BiLSTM or Transformer models	Published models limited to 5mCpG
	DeepPlant [68]	5mC in CG, CHG, CHH contexts	Deep-learning framework optimized for plant epigenomes	Poor support for animal-specific modification profiles
	Guppy/Megalodon/Dorado (ONT official) [18]	Model-dependent: 5mC, 5hmC, 6mA; selected Dorado models also support 4mC	Modification-aware model outputs; Megalodon additionally uses reference alignment	Performance constrained by pore chemistry version and training datasets

Note: Assay requirements differ: bisulfite-based methods can damage DNA, whereas antibody-enrichment methods have different sources of bias. Standard WGBS measures combined 5mC/5hmC; estimating 5hmC with oxBS-seq requires a matched BS-seq measurement. Nanopore tools infer selected modifications from native-DNA signals, with performance dependent on model, chemistry, sequence context, and coverage. The table reports capabilities demonstrated in the cited studies rather than all theoretically possible model extensions. Orthogonal validation should match the modification being tested. Traditional methods remain essential as orthogonal validation benchmarks for nanopore-predicted modification sites.

## Data Availability

No new data were created or analyzed in this study. Data sharing is not applicable to this article.
